# Genotypic characteristics of Chinese patients with BHD syndrome and functional analysis of *FLCN* variants

**DOI:** 10.1186/s13023-019-1198-y

**Published:** 2019-10-15

**Authors:** Keqiang Liu, Wenshuai Xu, Xinlun Tian, Meng Xiao, Xinyue Zhao, Qianli Zhang, Tao Qu, Jiaxing Song, Yaping Liu, Kai-Feng Xu, Xue Zhang

**Affiliations:** 1 McKusick-Zhang Center for Genetic Medicine, State Key Laboratory of Medical Molecular Biology, Institute of Basic Medical Sciences, Chinese Academy of Medical Sciences & Peking Union Medical College, Beijing, 100005 China; 2 Department of Pulmonary and Critical Care Medicine, Peking Union Medical College Hospital, Chinese Academy of Medical Sciences & Peking Union Medical College, Beijing, 100730 China; 3 Department of Dermatology, Peking Union Medical College Hospital, Chinese Academy of Medical Sciences & Peking Union Medical College, Beijing, 100730 China

**Keywords:** Birt-Hogg-Dubé syndrome (BHDS), *FLCN*, Mutation spectrum, Clinical manifestations, Minigene assay, Non-truncating mutation

## Abstract

**Background:**

Birt-Hogg-Dubé syndrome (BHDS) is an autosomal dominant disease featured by lung cysts, spontaneous pneumothorax, fibrofolliculomas and renal tumors. The causative gene for BHDS is the *folliculin* (*FLCN*) gene and more than 200 mutations have been reported in *FLCN*, mostly truncating mutations. The aim of this study is to better characterize the clinical features and mutation spectrum of Chinese BHDS patients and to systematically evaluate the effects of non-truncating mutations on mRNA splicing pattern.

**Methods:**

We enrolled 47 patients from 39 unrelated families with symptoms highly suggestive of BHDS after informed consent and detailed clinical data were collected. Exon sequencing followed by multiplex ligation-dependent probe amplification testing were applied for mutation screening. The effects of non-truncating mutations, including 15 missense mutations and 6 in-frame deletions, on mRNA splicing were investigated by minigene assays.

**Results:**

A total of 24 *FLCN* germline variants were found in 39 patients from 31 distinct families. Out of these patients, 100% (36/36) presented with lung cysts and 58.3% (21/36) had experienced spontaneous pneumothorax. Seventeen mutation carriers had skin lesions (47.2%, 17/36) and 9 (30%, 9/30) had kidney lesions including 8 with renal cysts and 1 with renal hamartoma. Among all detected variants 14 (58.3%, 14/24) were novel, including 11 variants classified to be pathogenic and 3 variants of uncertain significance. None of 21 non-truncating mutations changed the mRNA splicing pattern of minigenes.

**Conclusions:**

We found different clinical features of Chinese BHDS patients compared with Caucasians, with more lung cysts and pneumothorax but fewer skin lesions and malignant renal cancer. Chinese patients with BHDS also have a different mutation spectrum from other races. Non-truncating mutations in *FLCN* did not disrupt mRNA splicing pattern, in turn supporting the hypothesis that these mutations impair folliculin function by disrupting the stability of the FLCN gene product.

**Electronic supplementary material:**

The online version of this article (10.1186/s13023-019-1198-y) contains supplementary material, which is available to authorized users.

## Background

Birt-Hogg-Dubé syndrome (BHDS) is an autosomal dominant inherited disorder caused by germline mutations in the *folliculin* (*FLCN*) gene [1], which encodes a tumor suppressor protein. BHDS phenotype is characterized by fibrofolliculomas on the face, neck and upper torso, lung cysts, spontaneous pneumothorax, and increased risk for renal tumors. The symptoms of skin, pulmonary, and renal appear independently with high association, and BHDS patients can present with a single phenotype or a combination of any of them [2]. This combination contributes to the great variability in clinical features of BHD syndrome, making clinical diagnosis generally difficult. Clinical and genetic studies about BHDS were mainly conducted among Caucasian population in the US and Europe [3–5], which help to characterize the phenotype and mutation spectrum. A recent epidemiologic study with a large cohort in Japan found different phenotypes and *FLCN* mutational hotspots (c.1347_1353dupCCACCCT and c.1533_1536delGATG) in Japanese BHDS patients [6]. While, BHDS studies were rarely reported in China and most of them were case reports. Our previous study [7] reported a much lower prevalence of skin lesions (11.1%, 3/27) and a different *FLCN* mutation spectrum in Chinese BHDS patients compared with Caucasians. Further studies are warranted to better understand the clinical and genetic characteristics of Chinese BHDS patients.

Since *FLCN* was identified as the causative gene of BHDS in 2002 [1], over 200 mutations have been reported in BHDS patients according to the Human Genome Mutation Database (HGMD) [8]. A mutation hotspot of 1-bp deletion or duplication (c.1285delC/dupC) within the polyC (8) tract in exon 11 has been detected as the most common mutation in several different countries or races [3, 4, 6, 7]. Interestingly, the majority of reported *FLCN* mutations are protein-truncating mutations resulting in complete loss of function of the gene product [9], including frameshifts, nonsenses, canonical +/− 1 or 2 splice site mutations and single- or multi-exon deletions. By comparison, missense and in-frame mutations are less frequently observed in BHDS patients comprising only 12.6% (26/206) of all *FLCN* mutations in HGMD. In vitro experimental evidence has shown that most of the *FLCN* missense/in-frame mutations impaired folliculin tumor suppressor function by disrupting the stability of the protein [10]. However, emerging evidence shows that quite a portion of presumed missense mutations in other genes abolish protein function actually by disrupting the normal mRNA splicing pattern [11–13]. Whether this mechanism is involved in the pathogenicity of *FLCN* missense and in-frame mutations remains to be elucidated.

In the present study, we described the clinical and genetic features of a cohort of 39 Chinese BHDS patients from 31 distinct families, and systematically evaluated the effect of all *FLCN* missense and in-frame mutations reported in HGMD database on mRNA splicing pattern.

## Results

### Clinical features of Chinese BHDS patients

**Fig. 1:**
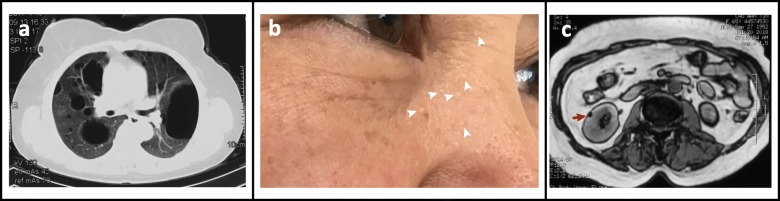
Representative manifestations in lung, skin and kidney observed in Chinese BHDS patients. **a** Chest CT showing multiple lung cysts in Patient 10. **b** Typical skin lesions (withe arrow heads) observed in Patient 7. **c** Abdominal MRI showing a renal cyst in patient 31 indicated by the red arrow

A total of 39 patients (37 Females, 2 Males) from 31 distinct families were diagnosed with BHDS by genetic testing (Table 1). The average age at diagnosis was 45.8 years (median = 48 y, range: 15–72 y, with one case unknown). Based on all information available, 85.7% (24/28) BHDS patients have a family history of lung cysts and/or spontaneous pneumothorax, and 2 (7.1%, 2/28) patients have a family history of renal tumor. In our cohort, 3 patients failed to follow-up, and all the rest 36 (100%, 36/36) patients have lung cysts (Fig. 1a), and 21 (58.3%, 21/36) have a history of pneumothorax. Seventeen (47.2%, 17/36) patients got cutaneous lesions (Fig. 1b) with multiple white or skin colored papules on the face, neck and/or feet. Renal impairment was observed in 9 (30%, 9/30) patients, including 8 with unilateral or bilateral renal cysts (Fig. 1c) and 1 with renal hamartoma. No malignant renal tumor was found in this cohort.

**Table 1:** Clinical characterizations and mutation analysis of Chinese BHDS patients

Patient/ family No.	Sex	Age	Family history	Spontaneous pneumothorax	Lung cysts	skin lesions	Kidney lesions	Smoking	Mutation (cDNA level)	Mutation (protein level)	Pathogenicity	Evidence
1	F	51	Y	Y	Y	Y	N	N	c.1287C > T^a^	p.His429=	VUS	PP3
2–1	F	72	Y	Y	Y	N	N	N	c.1283insG^a^	p.His429Profs*27	Pathogenic (Ia)	PVS1, PS3, PM1, PM2, PP3, PS1
2–2	F	49		Y	Y	N	N	N				
3	F	58	Y	N	Y	N	N	N	c.469_471delTTC	p.Phe157del	Pathogenic (II)	PS1, PS3, PM4, PP3
4	F	58	Y	N	Y	Y	Right renal cysts	N	c.1285dupC	p.His429Profs*27	Pathogenic (Ia)	PVS1, PS1, PS3, PM1, PM2
5–1	F	49	Y	Y	Y	N	N	N	c.1597C > T	p.Gln533Ter	Pathogenic (Ia)	PVS1, PS1, PS3, PM2, PP3
5–2	F	39		N	Y	N	N	N				
6	F	56	Y	Y	Y	Y	NA	N	c.1177-5_1177-3delCTC	–	Pathogenic (II)	PS3, PP3, PS1
7	F	58	N	N	Y	Y	N	N	c.780-12_780-3delTGTGTTCTCC^a^	–	VUS	PM2, PM6, PP3
8	F	54	Y	Y	Y	N	N	N	c.1579_1580insA	p.Arg527Glnfs*	Pathogenic (Ia)	PVS1, PS1, PM2, PP3
9–1	F	34	Y	Y	Y	N	Bilateral renal cysts	N	c.249 + 1G > A^a^	–	Pathogenic (Ia)	PVS1, PS3, PM2, PP3
9–2	F	57		N	Y	Y	N	12 pack-years				
10	F	61	Y	N	Y	Y	Bilateral renal cysts	N	c.1653_1654insTG^a^	p.Phe552Cysfs*4	Pathogenic (Ic)	PVS1, PM2, PP3
11–1	F	31	Y	Y	Y	N	N	N	c.1285dupC	p.His429Profs*27	Pathogenic (Ia)	PVS1, PS1, PS3, PM1, PM2
11–2	F	57		N	Y	N	N	N				
12	F	45	Y	Y	Y	N	Left renal cysts	N	c.250delG^a^	p.Gly84Alafs*46	Pathogenic (Ic)	PVS1, PM2, PP3
13	F	39	NA	NA	NA	NA	NA	NA	c.1285delC	p.His429Thrfs*39	Pathogenic (Ia)	PVS1, PS1, PS3, PM1, PM2
14–1	F	43	Y	Y	Y	N	N	N	c.1015C > T^a^	p.Gln339Ter	Pathogenic (Ic)	PVS1, PM2, PP3
14–2	M	22		N	Y	Y	NA	N				
14–3	F	15		N	Y	N	NA	N				
15	F	43	Y	N	Y	N	N	N	c.1533G > A	p.Trp511Ter	Pathogenic (Ic)	PVS1, PM2, PP3
16	F	33	Y	Y	Y	Y	N	N	c.1177-2A > C^a^	–	Pathogenic (Ic)	PVS1, PM2, PP3
17–1	F	48	Y	Y	Y	N	NA	N	c.1408delC	p.Cys471Alafs*3	Pathogenic (Ia)	PVS1, PS1, PM2, PP3
17–2	F	22		N	Y	N	NA	N				
18–1	M	51	Y	Y	Y	Y	N	N	c.929_930insTT^a^	p.Pro311Serfs*13	Pathogenic (Ic)	PVS1, PM2, PP3
18–2	F	28		Y	Y	Y	NA	N				
19	F	50	NA	NA	NA	NA	NA	NA	c.658C > T	p.Gln220Ter	Pathogenic (Ia)	PVS1, PS1, PM2, PP3
20	F	NA	NA	NA	NA	NA	NA	NA	c.1433-1G > T	–	Pathogenic (Ic)	PVS1, PM2, PP3
21	F	48	Y	N	Y	N	N	N	c.658C > T	p.Gln220Ter	Pathogenic (Ia)	PVS1, PS1, PM2, PP3
22	F	46	Y	Y	Y	N	N	N	△Exon1^a^	–	Pathogenic (Ia)	PVS1, PS3, PM1, PM2
23	F	45	Y	N	Y	Y	Left renal hamartoma	N	c.1285dupC	p.His429Profs*27	Pathogenic (Ia)	PVS1, PS1, PS3, PM1, PM2
24	F	35	N	Y	Y	N	N	N	c.1285dupC	p.His429Profs*27	Pathogenic (Ia)	PVS1, PS1, PS3, PM1, PM2
25	F	33	Y	Y	Y	Y	N	N	c.1285delC	p.His429Thrfs*39	Pathogenic (Ia)	PVS1, PS1, PS3, PM1, PM2
26	F	53	N	N	Y	Y	N	N	c.1301-2A > C^a^	–	Pathogenic (Ic)	PVS1, PM2, PP3
27	F	44	Y	Y	Y	Y	N	N	c.1285dupC	p.His429Profs*27	Pathogenic (Ia)	PVS1, PS1, PS3, PM1, PM2
28	F	50	Y	Y	Y	Y	Renal cysts	N	c.980_981insC^a^	p.Glu328Argfs*62	Pathogenic (Ic)	PVS1, PM2, PP3
29	F	44	Y	Y	Y	N	Bilateral renal cysts	N	c.1285dupC	p.His429Profs*27	Pathogenic (Ia)	PVS1, PS1, PS3, PM1, PM2
30	F	54	Y	Y	Y	Y	Right renal cysts	N	c.1227C > G^a^	p.Tyr409Ter	Pathogenic (Ic)	PVS1, PM2, PP3
31	F	66	Y	N	Y	Y	Bilateral renal cysts	N	c.282_290delATATATCAG^a^	p.Tyr95_Ser97del	VUS	PM2, PP1, PP3

^a^Novel mutation; *NA* not available, *VUS* variant of uncertain significance

### Germline *FLCN* variants detected in this cohort

Mutation screening by direct sequencing and MLPA analysis of the *FLCN* gene were performed in a total of 39 unrelated probands. Out of them, each of 31 probands was confirmed to carry a suspected disease-causing variant in *FLCN* (Table 1). The overall variant detection rate was therefore 79.5% (31/39). Altogether, 24 distinct variants were identified, including 21 (87.5%, 21/24) truncating variants (including nonsense, frameshift, putative splicing mutation and gross deletion) predicted to cause complete loss of function of folliculin, 2 in-frame small deletions and 1 synonymous variant. The mutational hot spot, a single duplication/deletion of cytosine in exon 11, was observed in 8 probands (6 c.1285dupC and 2 c.1285delC), which was the most frequent mutation in our cohort. Interestingly, a new form in this poly-cytosine tract, c.1283insG, was found in proband 2–1 and her affected daughter.

**Fig. 2:**
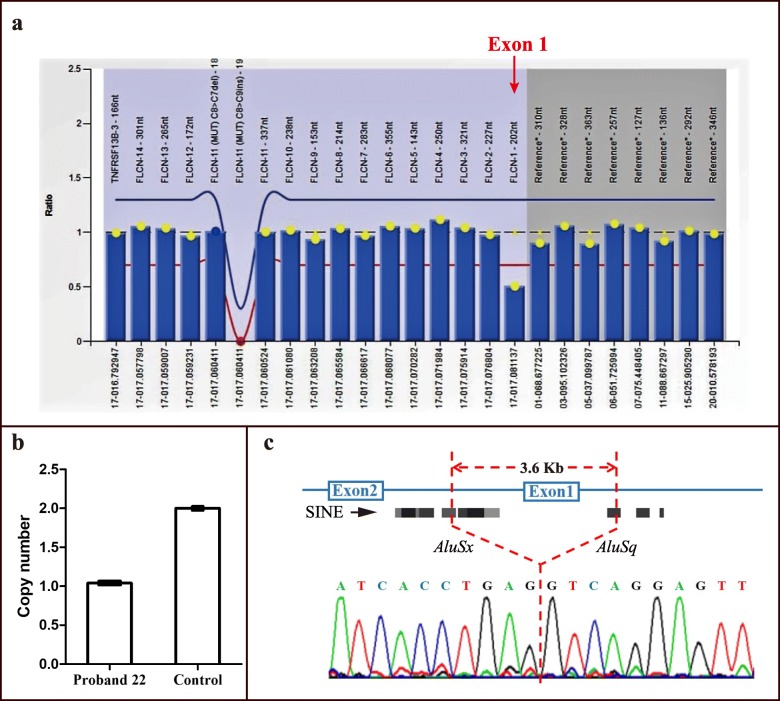
*FLCN* exon 1 deletion found in patient 22. **a** Exon 1 deletion was detected by MLPA. The X-axis shows the genomic positions of the probes and the Y-axis represents the signal ratio compared with control. The red arrow represents the heterozygous deletion of exon 1. **b** Quantitative real-time PCR for *FLCN* exon 1**.** Data were normalized with the copy number of a healthy control and experiments were performed in triplicates. **c** Sanger sequencing revealed a deletion of about 3.6 Kb encompassing *FLCN* exon 1. The deletion boundaries involved the repeat elements *AluSx* in intron 1 and *AluSq* in upstream of *FLCN*

Among the 24 germline variants detected in this study, 14 (58.3%, 14/24) were found to be novel variants. According to the ACMG/AMP 2015 guidelines [14], 11 novel variants were classified as pathogenic (Ia – Ic), including 5 frameshift, 2 nonsense, 3 canonical splice site variants and 1 gross deletion. The gross deletion at *FLCN* exon 1 (△Exon 1) was detected in proband 22 by MLPA and validated by genome DNA qPCR (Fig. 2a, b). Characterization of the breakpoints using long-range PCR showed that there was a deletion of 3.6 Kb encompassing the entire exon 1 (Fig. 2c). The gross deletion was flanked by a repeat element *AluSx* in intron 1 and an *AluSq* in the upstream region of *FLCN*.

**Fig. 3:**
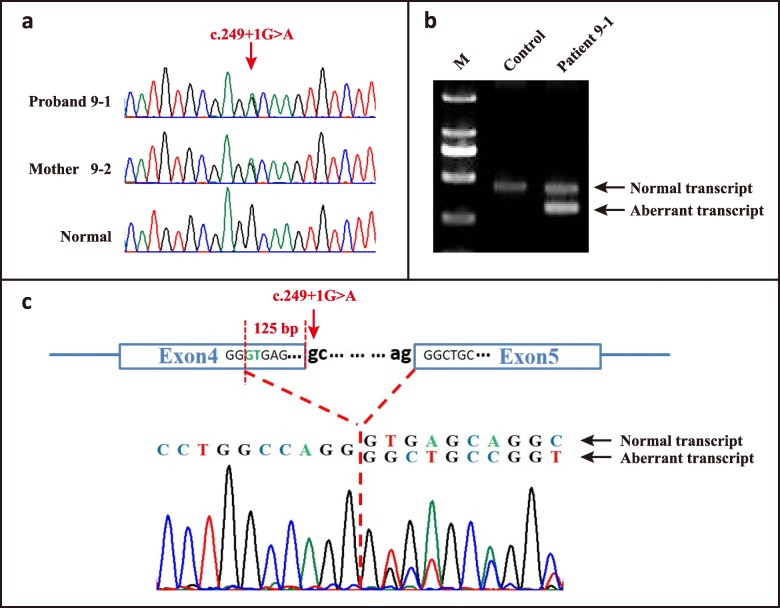
Mutation analysis for patient 9–1. **a** A splicing mutation c.249 + 1G > A was detected in patient 9–1 and her mother. **b** RT-PCR of mRNA from patient 9–1 yielded an extra shorter band compared with control, indicating an aberrant transcript. **c** Sequencing result of the RT-PCR products showed a partial deletion of 125 bp in *FLCN* exon 4, resulting from the activation of a cryptic splice site within exon 4 (shown in green)

Variant c.249 + 1G > A was found in proband 9–1 and her affected mother 9–2 (Fig. 3a), which was predicted to disrupt the canonical splice site. RT-PCR analysis of the mRNA from peripheral blood of the patient was performed. In the absence of the normal splice site, a cryptic splice site within exon 4 was adopted during mRNA splicing process, resulting in an aberrant transcript bearing a partial deletion of 125 bp in *FLCN* exon 4 (Fig. 3b). The resultant out-of-frame deletion in exon 4 led to a premature termination codon.

The other three novel variants (c.1287C > T (predicted as p.His429=), c.780-12_780-3delTGTGTTCTCC and c.282_290delATATATCAG (p.Tyr95_Ser97del)) were classified to be VUS. The 10-bp deletion in intron 7 (c.780-12_780-3delTGTGTTCTCC) was absent from gnomAD with a strong in silico prediction to disrupt the acceptor site by Human Splicing Finder and MutationTaster (data not shown). The in-frame deletion c.282_290delATATATCAG (p.Tyr95_Ser97del) was also not seen in gnomAD, and was predicted to be disease-causing by MutationTaster (data not shown). The c.1287C > T variant was not seen in East Asia but in other races according to gnomAD, of which the clinical significance was evaluated as likely benign in ClinVar. However, direct evidence for the pathogenicity of these 3 VUS was not obtained. Further analysis for the mRNA from these patients and protein stability test will help to classify these variants as pathogenic or benign.

### Missense/in-frame *FLCN* mutations do not affect minigene splicing pattern in vitro

**Fig. 4:**
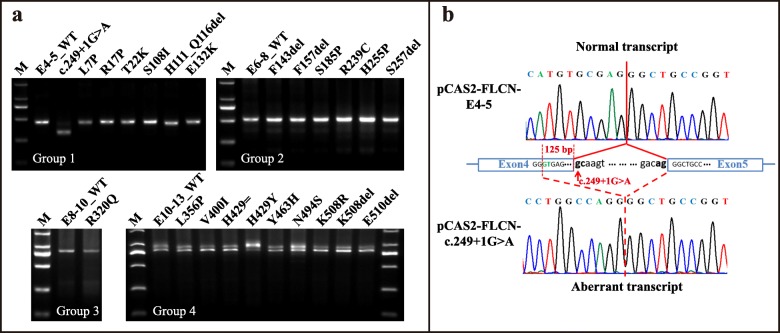
Splicing pattern evaluation by in vitro minigene assays. **a** Non-truncating mutations in *FLCN* were divided into four groups for splicing evaluation. None of these non-truncating mutations led to an abnormal transcript, compared with those of the wild-type minigenes. While, the positive control, variant c.249 + 1G > A, yielded a shorter transcript as expected. **b** Sanger sequencing of the aberrant transcript produced by c.249 + 1G > A showed that this mutation caused a partial deletion of 125 bp in *FLCN* exon 4, consistent with the in vivo results from patient 9–1

Coding exons of *FLCN* were divided into 4 groups to construct minigene expression vectors. A total of 15 missense mutations, 6 in-frame deletions documented in the public database and the c.1287C > T variant found in patient 1 in this study were selected for in vitro splicing pattern evaluation. In addition, the c.249 + 1G > A variant was also investigated, serving as a positive control. After direct-mutagenesis, wild-type and mutant minigene vectors were transiently expressed in HEK293T cells to analyze the mRNA splicing pattern. RT-PCR results showed that, in all of the four wild-type minigenes, corresponding *FLCN* exons were successfully included in the mature chimeric transcript as expected, which was confirmed by Sanger sequencing (data not shown). Interestingly, the wild-type pCAS2-FLCN-E10–13 minigene produced an additional transcript with exon 11 skipped (Fig. 4a, Group 4), which has previously been observed in normal human cDNA and the product of another reported minigene containing *FLCN* exon 11 [15]. Moreover, the positive control minigene carrying variant c.249 + 1G > A produced an aberrant transcript (Fig. 4a, group1 line 3). Sanger sequencing showed that the aberrant splicing product adopted a cryptic splice site within exon 10 causing a deletion of 125 bp (Fig. 4b), consistent with the in vivo results from patient 9–1 as shown in Fig. 3c.

The impact of these non-truncating mutations on splicing was determined by comparison of the transcripts obtained from the mutant constructs with the corresponding wild-type minigenes. Unexpectedly, none of these mutations led to an abnormal transcript of different size from respective wild-type products (Fig. 4a). Subsequent sequencing of each band of RT-PCR products confirmed the identity of splicing pattern between the wild-type and mutant minigenes (data not shown). Variants introduced into pCAS2-FLCN-E10–13 (group 4) also produced two transcripts as same as the wild-type produced. And gel electrophoresis did not show significantly increased ratio of exon 11 skipping in these mutant minigenes.

## Discussion

*FLCN* was first identified as the causative gene for BHDS in 2002 [1], making genetic testing an effective diagnosis method for this disease. While clinical and genetic studies of BHDS are rare in Asian population, especially in Chinese. In the present study, we reported a large Chinese BHDS cohort with 39 patients from 31 unrelated families diagnosed by genetic testing, including 37 female and 2 male patients. Much more female patients were observed in our patients, which is likely due to a selection bias because Peking Union Medical College Hospital (PUMCH) is the largest lymphagioleiomyomatosis referral center in China. The same selection bias has been reported in a Japanese study [16].

Respiratory system was the most frequently affected system in these BHDS patient as we reported before [7], with frequencies of almost 100 and 58.3% for lung cysts and pneumothorax respectively. Similarly, a recent epidemiologic study of Japanese BHDS patients reported that all except one family had lung cysts and 73.7% presented with episodes of pneumothorax in their cohort [6]. By comparison, lower prevalence of lung cysts (70–85%) was reported in Caucasian BHDS patients and only about one-third patients reported a history of spontaneous pneumothorax [4, 5, 17, 18]. Thus, it seems that pulmonary manifestations are more frequently observed in Chinese BHDS patients. What is worth mentioning is that the Caucasian patients from literature were all recruited through referrals from department of dermatology or urology. While, most patients in this study came to our clinic because of cysts in the lung. So, selection bias should be responsible, at least partially, for the different frequencies of pulmonary manifestations between Chinese and Caucasian BHDS.

BHDS affected individuals are reported to have a higher risk to develop renal tumors with a prevalence of 12–34% in Caucasian population [3–5, 18, 19] and about 34.8% (40/115) in Japanese patients over the age of 40 [6] . The most common histologic type of renal tumor in BHDS patients are chromophobe renal cell carcinomas and hybrid oncocytic neoplasms with features of chromophobe renal cell carcinoma and oncocytoma [20]. While, among 30 patients with available data in our cohort, there were 8 patients with unilateral or bilateral renal cysts and 1 with renal hamartoma, but no renal malignancy. Similar findings have been reported in our previous studies, in which no malignant renal tumor was diagnosed in 27 probands, except for 1 with hamartomas and 4 with renal cysts [7]. Despite potential selection and non-response bias in these two studies, malignant renal tumor was rarely detected in these Chinese *FLCN* mutation carriers, which strongly suggests a lower prevalence of renal malignancy in Chinses BHDS patients.

More interestingly, about half of the patients (47.2%, 17/36) in this cohort were found to have cutaneous lesions, much more than that we reported before (11.1%, 3/27) [7]. Relatively small sample size and more clinical awareness in China might be responsible for the inconsistency. A similar frequency (48.7%, 76/156) of skin lesions was documented in Japanese BHDS patients [6]. In contrast, cutaneous findings were reported to be the most common symptoms among Caucasian patients with a percentage of around 90% [4, 5, 17]. So, there was much less skin manifestation observed in Chinese BHDS individuals compared with Caucasians. Collectively, this study showed that Chinese BHDS patients have different clinical features from Caucasians, with more pulmonary manifestations but fewer skin lesions and renal malignancy.

In our previous study, 14 out of 20 variants found in a Chinese BHDS cohort were novel [7], indicating a different mutation spectrum from that of Caucasians. And in the present study, as a follow-up study, more than half (52.4%, 11/21) of the 21 detected different variants have never been reported. Collectively, these two studies demonstrated the variability of *FLCN* mutation spectrum between Chinese and other races. A previously reported mutational hot spot, c.1285dupC/delC, is also the most frequent mutation observed in these two studies, with a frequency (28.3%, 15/53) comparable to that of Japanese patients (28.3%, 34/120) [6]. However, the other two mutational hot spots c.1347_1353dupCCACCCT (p.Val452ProfsX6) and c.1533_1536delGATG (p.Trp511X) reported in Japanese were not observed in our cohort. The only one mutation observed in more than 5 patients is c.1285dupC. We compared the pulmonary, cutaneous and renal manifestations between c.1285dupC carriers and other patients. No association was observed between this mutation with any clinical features in our cohort.

Gross intragenic rearrangements of *FLCN* were less frequently reported. To our knowledge, the Exon 1 deletion identified in this study is the second gross deletion reported in Chinese BHDS patients. Unlike the first reported △Exon 8 mutation [7], this deletion is located in the 5′-untranslated region. Long-range PCR and bidirectional sequencing revealed a 3.6 Kb deletion encompassing the entire exon 1 flanked by the repeat elements *AluSx* in intron 1 and *AluSq* in the upstream region of *FLCN*. Benhammou et al. [21] reported several BHDS families carrying deletions involving exon 1 and found that the genomic sequences of this region contain a significantly higher number of *Alu* elements than the rest of the entire *FLCN* gene, which in turn might be responsible for the deletions due to unequal crossover mediated by these *Alu* elements. In vitro luciferase reporter assay indicated that this region contains the putative *FLCN* promoter [21], deletion of which will significantly reduce the expression of *FLCN*, supporting the pathogenicity of this △Exon 1 mutation.

The synonymous variant, c.1287C > T (p.His429=), found in patient 1 was classified as VUS and was observed in gnomAD with the highest frequency of about 1e^− 4^ in Ashkenazi Jewish. Besides, the clinical significance of this variant was evaluated as likely benign in ClinVar by multiple submitters with no conflicts. Moreover, minigene assay showed that this variant did not affect mRNA splicing. Therefore, this variant might be not responsible for the phenotypes in patient 1. Further investigations including phenotypic validation and application of other mutation detection tool are needed.

As a putative tumor suppressor, the vast majority of reported *FLCN* mutations are truncating mutations including nonsenses, frameshifts, splice site mutations and large deletions/duplications, which will result in truncated protein or absent of mRNA by nonsense-mediated decay. By contrast, variants causing amino acid substitution or in-frame insertion/deletion are much infrequently reported. The *FLCN* mutation database established by Lim and colleagues [9] reported that missense mutations only count for 8.6% (6/70) of all *FLCN* mutations reported at that time. In a large Japanese BHDS cohort, missense/in-frame mutations counted for 9.2% of all patients with germline mutation. In addition, only 1 missense (c.1067 T > C, p.Leu356Pro) [7] and 1 in-frame deletion (c.469_471delTTC, p.Phe157del identified in this study) were observed in our cohort of 53 Chinese patients. Unlike truncating mutations, the pathogenicity of missense/in-frame mutations are relatively difficult to determine, which requires co-segregation of the variants with disease status in relatively large kindred and solid functional evidence. Previously, researchers transiently expressed folliculin in wild-type form or mutant form carrying specific missenses/in-frame deletions in FTC-133 cell line, and found that most of these non-truncating mutations significantly disrupted the stability of folliculin protein [10]. However, by directly inserting the full-length *FLCN* cDNA into expression vector, this strategy did not rule out the possibility that these mutations might cause loss of gene function actually by affecting the mRNA splicing process [11–13].

To investigate the potential effects of *FLCN* non-truncating mutations on mRNA processing, we analyzed the splicing patterns of all 21 reported non-truncating mutations (15 missenses and 6 in-frame deletions) as well as the c.1287C > T variant (predicted as p.His429=), in an in vitro system based on the splicing reporter minigene [22]. All wild-type minigenes produced expected chimeric transcripts, while the c.249 + 1G > A variant, used as a positive control, produced a same aberrant transcript as shown in vivo. To a certain degree, it reflected the reliability of this splicing system. However, none of 21 non-truncating mutations changed the splicing pattern of minigenes. Moreover, normally spliced transcripts bearing the corresponding substitutions or deletions were observed by direct sequencing. These results denied the hypothesis that *FLCN* missense/in-frame mutations cause BHDS phenotypes through affecting mRNA splicing. And, with this observation, the hypothesis of disrupted folliculin stability seems more reasonable. However, we should also note that the in vitro minigene assay may not fully represent the splicing regulatory machinery involved in BHDS affected tissues.

There are some limitations in our study. Firstly, data missing is a major problem, especially for renal and cutaneous examinations. Data about kidney examination in 9/39 patients are not available, which make it difficult to correctly define the prevalence of renal impairments. Because skin lesions like fibrofolliculomas are generally neither painful nor pruritic, most patients were reluctant to accept invasive biopsy. Thus, only one patient was diagnosed with typical skin lesions by histological confirmation. Secondly, most of the patients were recruited from a single center, Department of Pulmonary and Critical Care Medicine, PUMCH, which is a referral center for rare pulmonary diseases in China. Patient were likely to be referred to our hospital when they have lung cysts on radiological scans. So, the finding of 100% lung cysts in this cohort should be used carefully, due to obvious selection bias. However, in these genetically confirmed BHDS patients, cutaneous involvements and renal tumors were much less frequently detected than in those reported in Caucasians. Given that skin, pulmonary, and renal symptoms appear independently in BHDS, our data even incomplete still support that Chinese BHDS patients have fewer skin lesions and kidney tumors.

## Conclusions

In the present study, we reported the clinical symptoms and *FLCN* variants in 39 Chinese patients with BHDS from 31 distinct families. The clinical features of Chinese BHDS patients were different from that of Caucasians, with more lung cysts and pneumothorax but less skin and kidney lesions. Moreover, 14/24 novel *FLCN* mutations were reported in this cohort, indicating a different mutation spectrum in Chinese from other races. In addition, by systematic in vitro minigene assays, we found that non-truncating mutations in *FLCN* did not disrupt mRNA splicing pattern, which in turn supports that these mutations cause disease by disrupting folliculin protein stability.

## Subjects and methods

### Study population

From February 2017 to February 2019, patients with unexplained multiple cysts and/or spontaneous pneumothorax visiting Peking Union Medical College Hospital (PUMCH) were screened for suspected BHDS. A total of 47 patients from 39 unrelated families were enrolled in this study and received genetic testing following the inclusion criteria proposed by the European BHD Consortium [23]. More concretely, patients with the following conditions were highly suspected of having BHDS: (1) having multiple lung cysts, especially bilaterally and basally located, or spontaneous pneumothorax with no apparent cause; (2) having a family history of cystic lung disease, pneumothorax or familial kidney cancer; (3) with any combination of unexplained lung cysts/pneumothorax, skin lesions like fibrofolliculomas or trichodiscomas confirmed by dermatologists, and nephropathy. Chest CT were applied to scan for lung impairments. Other diffuse cystic lung diseases, like lymphagioleiomyomatosis, light chain deposition disease, amyloidosis, infectious pneumocystis, tuberous sclerosis, lymphoid interstitial pneumonia and pulmonary Langerhans cell histiocytosis were excluded by high-resolution computed tomography. Skin examination was carried out in most patients and skin lesions were diagnosed by an experienced dermatologist. Renal involvements were detected by renal ultrasonography, abdominal CT scans and/or MRI. The study was approved by the Institutional Review Board committee at PUMCH.

### Mutation screening of *FLCN*

Genomic DNA was extracted from peripheral blood of the patients. Direct sequencing for all coding exons (exon 4–14) of *FLCN* and flanking sequences was performed as previously described [7]. Sequencing traces were analyzed using the CodonCode Aligner Software (CodonCode Aligner Corporation; Centerville, MA, USA) and variants nomenclature was described according to the transcript reference NM_144997.6. In addition, multiplex ligation-dependent probe amplification (MLPA) analysis was conducted following the manufacture’s instruction to screen potential gross rearrangement in patients with no pathogenic mutation identified by direct sequencing, using a commercial MLPA kit (SALSA® P256-B2 FLCN, MRC-Holland; Amsterdam, The Netherlands). Real-time quantitative PCR (qPCR) of genome DNA was carried out to verify the gross deletion detected by MLPA analysis as described before [24]. Subsequently, the deletion breakpoints were characterized by long-range PCR and Sanger sequencing. All variants were classified as (likely) pathogenic, variant of uncertain significance (VUS), or (likely) benign following the American college of medical genetics and genomics (ACMG) / Association of molecular pathology (AMP) 2015 guidelines [14].

### Generation of minigene constructs

**Fig. 5:**
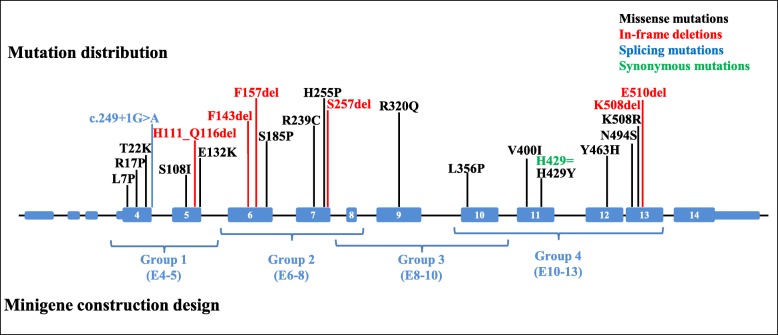
Schematic diagram describing the distribution of mutations analyzed within *FLCN*. The missense and in-frame mutations are distributed across the folliculin protein, rather than gathering in a specific functional domain. Exons harboring these mutations were divided into 4 groups for minigene construction

To investigate the potential effect of non-truncating mutations on *FLCN* transcript splicing pattern, a series of minigene assays were performed using the expression minigene plasmid pCAS2, which is modified based on the backbone of the mammalian expression vector pcDNA3.1(−) [22]. Previously reported pathogenic *FLCN* missense and in-frame mutations in HGMD database were all reviewed. A total of 19 missense and 6 in-frame mutations were recorded in HGMD at the beginning of this study, which are distributed across all coding regions rather than restricted to a specific domain of the folliculin protein (Fig. 5). All of these mutations except those affecting the initiation codon or located in the last exon, as well as the predicted synonymous variant detected in patient 1 (c.1287C > T, p.His429=), were investigated using this in vitro splicing system. Besides, the canonical splice site variant c.249 + 1G > A found in this study was also evaluated, which is located at the splice donor site and serving as a positive control of this system. Exons harboring these mutations were divided into 4 groups (Fig. 5 and Additional file 1). DNA fragments encompassing corresponding exons with flaking intronic sequences were amplified from the genomic DNA of a healthy volunteer and recombined into the pCAS2 vector using an In-Fusion HD Cloning Kit (Takara; Kusatsu, Shiga, Japan) according to the user manual. The resultant wild-type minigenes were named as pCAS2-FLCN-E4–5, pCAS2-FLCN-E6–8, pCAS2-FLCN-E8–10 and pCAS2-FLCN-E10–13, respectively. All mutations were introduced into corresponding minigenes using site-directed mutagenesis strategy. Primers for DNA fragments amplification and mutagenesis are available in Additional file 1. The resultant wild-type and mutant minigene plasmids were confirmed by Sanger sequencing.

### Cell culture and plasmid transfection

HEK293T cells were grown in a 5% CO_2_ incubator at 37 °C in Dulbecco’s modified Eagle’s medium (Union Cell Resource Center; Beijing, China) supplemented with 10% fetal bovine serum (Gibco; Grand Island, NY, USA) and 1x antibiotics (Life technologies; CA, USA). Cells were seeded at 80% confluence in a 6-well plate 12 h before transfection. Respective wild-type and mutant minigenes for each group were transfected into HEK293T cells at a concentration of 2.5 μg DNA per well using Lipofectamine 3000 Transfection Reagent (Invitrogen; CA, USA). Forty-eight hours after transfection, cells were lysed for transcript analysis.

### RNA extraction and RT-PCR analysis

For transcript analysis of the c.249 + 1G > A variant, total RNA from peripheral blood of patient 9 was extracted using TRIzol LS Reagent (Invitrogen; CA, USA) according to the manufacturer’s protocols. cDNA was synthesized using PrimeScript RT Master Mix (Takara; Kusatsu, Shiga, Japan). PCR was performed with primer pair FLCN-rt-F (5′-GCTGAGTGTCAGTGTGACCAC-3′) and FLCN-rt-R (5′-CACGGCCAGGGCAGACCTC-3′) spanning the junctions of exon 2/3 and exon 5/6, respectively. The resultant product was visualized by gel electrophoresis and analyzed by further Sanger sequencing.

For minigene assay, cells were washed twice with PBS 48 h after transfection. Total RNA was extracted using a standard procedure with TRIzol (Invitrogen; CA, USA) and chloroform. RT-PCR was performed immediately after RNA extraction with respective PCR primer pairs for each minigene group (primer sequences available in Additional file 1). Splicing products of each minigene were visualized by gel electrophoresis and analyzed by further Sanger sequencing.

## Supplementary information

**Additional file 1:** Primer sequences for minigene construction, mutagenesis and RT-PCR. (XLSX 15 kb)

## Data Availability

The datasets supporting the conclusions of this article are included within the article and its additional file.

## Abbreviations

ACMG: American college of medical genetics and genomics
AMP: Association of molecular pathology
BHDS: Birt-Hogg-Dubé syndrome
HGMD: Human genome mutation database
MLPA: Multiplex ligation-dependent probe amplification
PUMCH: Peking Union Medical College Hospital
VUS: Variant of uncertain significance
